# Wheeled Mobile Robots: State of the Art Overview and Kinematic Comparison Among Three Omnidirectional Locomotion Strategies

**DOI:** 10.1007/s10846-022-01745-7

**Published:** 2022-10-24

**Authors:** Luigi Tagliavini, Giovanni Colucci, Andrea Botta, Paride Cavallone, Lorenzo Baglieri, Giuseppe Quaglia

**Affiliations:** Department of Mechanical and Aerospace Engineering, Politecnico di Torino, Corso Duca degli Abruzzi 24, Turin, 10129 Italy

**Keywords:** Wheeled mobile robots, Kinematic models, Velocity space analysis, 4WD robots, 3WD robots, Swerve-drive robots

## Abstract

In the last decades, mobile robotics has become a very interesting research topic in the field of robotics, mainly because of population ageing and the recent pandemic emergency caused by Covid-19. Against this context, the paper presents an overview on wheeled mobile robot (WMR), which have a central role in nowadays scenario. In particular, the paper describes the most commonly adopted locomotion strategies, perception systems, control architectures and navigation approaches. After having analyzed the state of the art, this paper focuses on the kinematics of three omnidirectional platforms: a four mecanum wheels robot (4WD), a three omni wheel platform (3WD) and a two swerve-drive system (2SWD). Through a dimensionless approach, these three platforms are compared to understand how their mobility is affected by the wheel speed limitations that are present in every practical application. This original comparison has not been already presented by the literature and it can be used to improve our understanding of the kinematics of these mobile robots and to guide the selection of the most appropriate locomotion system according to the specific application.

## 1 Introduction

In the last decades, the global population has experienced an unprecedented decrease in fertility and mortality rates leading to a widespread population ageing. Such phenomenon affects the everyday life of the individuals and forces government and private organizations to seek solutions against the increasing demand for health care, housing, caregiving, and social security [1, 2]. The pandemic emergency, caused by Covid-19, has also highlighted the fact that, in those cases where patients may be endangered by the closeness to other people, it could be better to free some activities from the presence of human operators, especially in hospitals, geriatric wards and hospices [3]. Although human staff cannot be replaced entirely (e.g., for complex operations and companionship), properly conceived and instrumented mobile robots can perform basic assistance like blood pressure, temperature, and oxygen saturation measurements, patient monitoring, triage activities, delivering of lightweight medical products and documents or remote presence. In the last decades, many researchers in the robotic field addressed the theme of assistive robotics, developing

Luigi Tagliavini, Giovanni Colucci, Andrea Botta, Paride Cavallone, Lorenzo Baglieri and Giuseppe Quaglia contributed equally to this work.

✉ Luigi Tagliavini
luigi.tagliavini@polito.it

Giovanni Colucci
giovanni_colucci@polito.it

Andrea Botta
andrea.botta@polito.it

Paride Cavallone
paride.cavallone@polito.it

Lorenzo Baglieri
lorenzo.baglieri@polito.it

Giuseppe Quaglia
giuseppe.quaglia@polito.it

[1] Department of Mechanical and Aerospace Engineering, Politecnico di Torino, Corso Duca degli Abruzzi 24, Turin 10129, Italy several mobile robotic platforms conceived to help weak or non-self-sufficient subjects [4–7]. Moreover, mobile robots are widely adopted in the exploration of hazardous environments like space [8], biological or chemical contaminated environment [9], mine clearance [10], search and rescue [11, 12] and military operation. Other application fields for mobile robotics are logistic of warehouse and industries and transportation of heavy load in industrial environment [13], precision agriculture [14–16] and domestic environment for entertainment [17], education [18, 19], household or as a personal assistant [20–22]. According to their locomotion system, mobile robots can be classified into the following major categories [23]:

1. Land-based
    (a) Wheeled mobile robot (WMR)
    (b) Walking (or legged) mobile robot
    (c) Tracked slip/skid locomotion
    (d) Hybrid
2. Air-based
3. Water-based
4. Other

In the present document, the attention is focused on wheeled mobile robots (WMR) because of their central role as robot locomotion architecture [23]. The prevalent adoption of wheels in mobile robots design can be justified by their simplicity in design, modelling, construction and programming, especially when moving on flat or non-rugged terrain. Moreover, during motion on these terrains, WMR is characterized by lower energy consumption compared to tracked or legged robots and in most cases, there is no need for complex balancing systems. Wheeled mobile robots can be remote-controlled platforms or autonomous systems. The manual remote control is mainly adopted in hazardous environments to reduce the risk on human operators, while autonomous mobile robots are mostly adopted to perform repetitive and time-consuming tasks such as material and supplies transportation, inspections of structures and machines, households, etc. A robot is considered autonomous when it has the ability to determine the actions to be taken to perform a required task without external human operators. Due to their interdisciplinary nature, many classifications can be made for mobile robots. For example, locomotion, perception, cognition, and navigation are four of the most important aspects of an autonomous mobile robot [23].

In the following subsections, a brief state-of-the-art analysis of the relevant assosieted technologies is presented. Later, in Sections 2 and 3 three of the most commonly adopted omnidirectional locomotion strategies are analysed. In Section 2 the kinematic models are derived, while in Section 3 the kinematics of these platform are compared in terms of allowed velocity constraints under maximum wheels speed limitation. This velocity analysis is proposed to investigate how these platforms can achieve omnidirectional mobility andto provide a tool to select the most appropriate locomotion system for a specific application. For this reason, the following state-of-the-art overview mainly investigates the different locomotion architectures, while for the other crucial aspects, such as perception, control and navigation, the interested reader is addressed to more detailed works.

Regarding the platform's locomotion, as early as 1996, Campion et al. proposed a classification for mobile platforms in five categories [24] based on kinematics and dynamics properties of the respective mathematical models. Among such categories, they recognize a single omnidirectional class of robots (i.e. robots able to exhibit translational motions decoupled from the orientation of the perform [25]) and four variously constrained other classes, each one characterized by different actuation strategy. These five classes are here summarized and some examples are presented in Table 1:

- **Type I:** omnidirectional robots with no steering wheels. This class of robots exploits special designed wheels such as mecanum wheel [26–28], universal wheels, also known as omni wheels [29], orthogonal wheels [30], spherical/ball wheels [31, 32], to achieve omnidirectional mobility in the plane of motion. Thus, these platform can follow plane-trajectories with translational velocities decoupled from the angular ones. They have full mobility in the plane, which means that they are able to move in any direction without reorientation. In literature, this class of robots has been widely explored. On this subject, Taheri et al. proposed in 2020 a detailed and comprehensive review [33]. Because of their full-in-plane mobility, two of the most commonly adopted omnidirectional platform are included in the kinematic analysis and comparison provided in Sections 2 and 3.
- **Type II:** robots with no steering wheels but either one or several fixed wheels with a common axle. The common axle restricts the mobility of the robots to a 2D plane. Among this class of platforms, the differential drive locomotion is the most commonly adopted [34–36]. These platforms are characterized by two traction wheels that can rotate around a common axis. The wheel's angular velocity can be controlled independently. The instantaneous center of rotation always lies on the traction wheels common axis. Thus, these platform can move in the direction perpendicular to common axis and rotate around a vertical axis. Even if no sideways motion is allowed, this locomotion system is the most commonly adopted in wheeled mobile robot due to its simplicity of construction and control.

**Table 1** Pros and cons of the most commonly adopted locomotion systems

| Type | Pros | Cons |
|---|---|---|
| Type I: Omnidirectional robot based on mecanum wheels: | | |
|  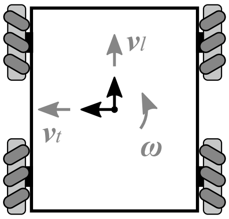 | Simple control | Discontinuous contact with the ground |
| | Omnidirectional mobility with fast response to turn | High sensitivity to floor condition |
| | Simple setup | High uncertainty and slippage with small control errors |
| | Low cost | Accuracy construction strongly affects performances |
| | Lightweight | Payload limitation |
| | | Actuation redundancy |
| Type I: Omnidirectional robot based on omni wheels: | | |
|  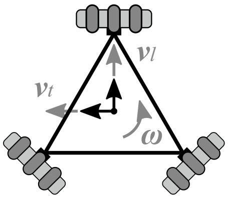 | Simple control | Discontinuous contact with the ground |
| | Omnidirectional mobility | High sensitivity to floor condition |
| | Simple setup | High uncertainty and slippage with small control errors |
| | Low cost | Accuracy construction strongly affects performances |
| | Lightweight | Payload limitation |
| | | Actuation redundancy |
| Type I: Omnidirectional robot based on spherical wheels: | | |
|  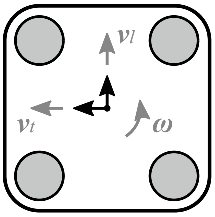 | Continuous contact with the ground | High uncertainty and slippage with small control errors |
| | High controllability | Accuracy construction strongly affects performances |
| | Omnidirectional mobility with fast response to turn | Mechanical complexity |
| | Little slippage | Actuation redundancy |
| | Little sensitivity to floor condition | |
| Type II: Differential-drive robot: | | |
|  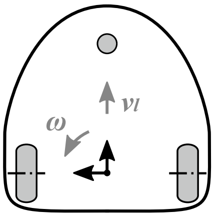 | Continuous contact with the ground | Reduced mobility: no transversal velocity allowed |
| | High payload | Vibration caused by castor wheels |
| | High efficiency | |
| | Little sensitivity to floor condition | |
| Type III: Wheeled robot with coupling steering mechanism: | | |
|  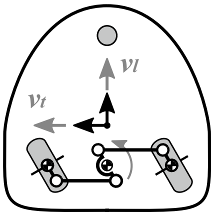 | Continuous contact with the ground | Reduced mobility: no angular velocity allowed during generic translations |
| | High payload | Vibration caused by castor wheels |
| | High efficiency | |
| | Little sensitivity to floor condition | |
| Type IV: Car-like robot: | | |
|  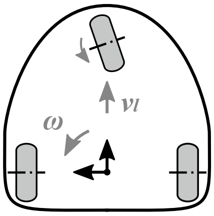 | Continuous contact with the ground | Reduced mobility: no transversal velocity allowed |
| | High payload | Multiple steering wheels are subjected to |
| | High efficiency | Ackerman constraint |
| | Little sensitivity to floor condition | |

**Table 1** (continued)

| Type | Pros | Cons |
|---|---|---|
| Type V: Pseudo-omnidirectional robot based on swerve-drive systems: | | |
|  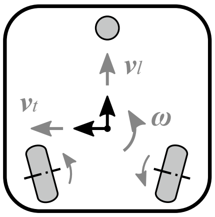 | Continuous contact with the ground<br>High mobility<br>High payload<br>High efficiency<br>Little sensitivity to floor condition | Extra-steering axis<br>Non-holonomic constraint<br>Pseudo-omnidirectional mobility<br>(time for reconfiguration with sharp corner trajectory) |

- **Type III:** robots with no fixed wheels and at least one steering wheel. Platform with more than one steering wheel may be classified as Type III only if the wheels point at the same direction. Thus, the mobility of these robots is restricted to a 2D plane. Some examples of platform within this class of robot is are presented in the works [37–39].
- **Type IV:** robots with one or several fixed wheels on a common axle and also one or several steering wheels. The steering wheels must not be located on the common axle of the fixed wheels. Moreover, if more than one steering wheels is adopted their orientations must be controlled according to a kinematic constraints (e.g. Ackerman constraint). Mobility is restricted to a 1D plane determined by the orientation angle of the steering wheel. Examples of this type are the tricycle, the bicycle, and the car-like WMR ([40–42].
- **Type V:** robots with no fixed wheels, but at least two independent steering wheels. If there are more than two steering wheels, then their orientation must be coordinated in two groups. This kind of locomotion architecture can be exploited adopting several actuation strategies. For example, a generic velocity twist can be achieved with a proper reconfiguration of the steering angles. On the contrary, when the wheel axes lie on the same line, the platform mobility is the same as Type II (differential drive locomotion), while in the other cases the mobility of the platform, at a defined steering angle configuration, is restricted to a 1D plane, because of a set of non-equal steering angles defines the position of the instantaneous center of rotation. For these interesting characteristics, an example of this class of robot is included in the kinematic analysis and comparison provided in Sections 2 and 3.

Regarding robot's locomotion systems, a more recent study [43] in 2018 by Gao et al. presents simple and reliable mathematical models for types I and II, while in 2015, Safar proposed a further classification of omnidirectional WMR according to their kinematics, distinguishing between holonomic and non-holonomic systems [44]. Anyway, all categories presented by Campion et al. have been exploited in the past. In particular, two classes of robots have been studied: the robots owning two fixed driven wheels, also known as differential-drive platforms and the omnidirectional platform based on mecanum/universal wheels. A reason for that can be found in their simplicity of construction and control. In fact, once the technology of the specially designed wheels improved, the design of these kinds of platforms is substantially simplified by the absence of steering axes resulting in simplified cable management and mechanical design. Nevertheless, the adoption of differential-drive locomotion systems implies a strong mobility limitation (the linear velocity of the platform is constrained along the longitudinal axis, i.e. the axis perpendicular to the actuated wheels' common axle), while the performances of mecanum/universal based platforms are strongly influenced by the reduced size of the passive rollers, which limits the ability in carrying heavy loads, and by the discontinue contact with the ground, which causes vibrations especially on uneven terrain [45, 46]. Moreover, a study by Adamov in 2018 [47] shows that the construction accuracy of Mecanum wheels affects the navigation of an omnidirectional platform (in that case a KUKA YOUBOT) and they proposed an improved algorithm to increase the accuracy of odometric navigation. In Table 1, the most adopted locomotion systems are reported and compared.

Another fundamental subsystem, especially for autonomous robots, is the perception unit. In robotics, the term perception describes the ability of the robot to perceive and comprehend the surrounding environment [48]. This is achieved by means of sensors, sensory data processing and data representation (environment modeling). Perception systems enable the robot with positioning and localization capability and provide data for mapping, planning and object recognition tasks. Finally, the latest advances in sensoring and artificial intelligence are being used in speech recognition systems to explore alternative human-machine interaction emulating human capabilities [23]. Some practical examples of robotic perception subareas are obstacle detection [49, 50] and recognition [51, 52], semantic place [53, 54], activity [55] and terrain classification [56], 3D environment representation [57], gesture and voice recognition [58, 59], road [60], vehicle [61, 62], pedestrian detection [63], object tracking [64], human [65] and environment change detection [66].

The control system represents a fundamental module of a robotic system. The information about the environment and the state of the robot, provided by the perception system, is processed to evaluate the appropriate commands for the actuation system. Back in 2001, Chung et al. inquired about mobile robot position kinematics and control [67]. Later in 2010, Chwa developed a recursive linearization control law for a particular robot model [68]. Also, over-actuated robots have been widely studied, with particular attention to non- or quasi-holonomic constraints implications [69, 70], and about the difficulties aroused by the control of an over-abundant set of actuators [71]. One of the objectives of control is to track the robot pose. The way in which the pose error is computed and tracked (for example feedback frequency, proportional and derivative gains, and so on) differentiates the type of control strategies that may be implemented [72].

Finally, navigation skills are essential for autonomous mobile robots. The goal of this module is to provide the robot with the capability to move from one place to another in full autonomy. To accomplish such a task, the mobile robot must rely on perception, localization, cognition and motion control. In most of the cases, the direct path from an initial pose to a final goal in not possible because of the presence of obstacles, both static (e.g. wall, furniture, building columns, ecc..) and dynamic (e.g. person, animals or other machines in movement inside the same environment). For this reason, motion planning techniques must be used. The complete execution of a navigation task can be divided into three major subsets: map generation, computation of a collision-free trajectory and moving along the planned trajectory while avoiding collision with obstacles. According to [33], navigation approaches are classified into three categories: global methods (e.g. roadmap methods [73, 74], cell decomposition methods [75, 76] and potential field methods [77, 78]), local (e.g. Vector Field Histogram (VFH) [79], VFH+ [80], VFH* [81], Virtual Force Field (VFF) [82], Potential Fields [83], Traversability Field Histogram (TFH) [84], Nearness Diagram (ND) [85], Elastic Band [86], Obstacle-restriction approaches [87] and dynamic window [88]) and hybrid approaches [89, 90, 93].

## 2 Platforms' Kinematics

In this section the kinematic models of three of the most commonly adopted omnidirectional locomotion strategies are derived. The pose of a robotic platform with respect to (w.r.t in the following) a space reference frame (r.f. in the following) $\{s\}$ can be represented by the configuration matrix ${}^{s}\mathbf{T}_b \in SE(2)$ or by the configuration vector $[\gamma_b, x_b, y_b]^T$ where $x_b$ and $y_b$ are the position coordinates of $\{b\}$ expressed in the $\{s\}$ frame and $\gamma_b$ is the rotation around the $z$-axis of $\{b\}$ expressed in the $\{s\}$ frame. The motion act of the mobile robot can expressed through the 3-dimensional velocity twist $\mathbf{V}_b = [\dot{\gamma}_b, \dot{x}_b, \dot{y}_b]^T$. Wheeled mobile robots employ either conventional wheels, that do not enable sideways sliding, or wheels that allow sideways sliding through the use of rollers around the rim of the wheels, e.g. mecanum/omni wheel based robots. For a non-holonomic mobile robot, like differential drive platforms, the space of feasible chassis velocities is two-dimensional because the robot cannot slide sideways. For an omnidirectional robot, the chassis can move in any direction, while rotating around a vertical axis, thus the velocity space is three-dimensional.

In order to achieve an omnidirectional motion in plane, the center of the $i$-th wheel, located at ${}^{b}\mathbf{p}_i$ w.r.t. the body frame $\{b\}$, should be able to move in any direction in the plane of motion. In Fig. 1, a simple velocity analysis is presented for three types of wheel: a mecanum wheel (a), an omni wheel (b), and a conventional wheel (c).

The specially designed mecanum wheels and omni wheels are characterized by the presence of small rollers, radius $r_r$, mounted at a certain angle around the rim of the wheel. These passive rollers enable a free-sliding motion along the direction perpendicular to the axis of the roller in contact with the ground. Under the assumption of pure rolling motion, given a linear velocity $\dot{\mathbf{p}}_i$ at the center of the $i$-th wheel, it is possible to decompose the desired velocity into two components: one along with the driving direction and the other along the free-sliding direction. The component along the driving direction is directly related to the angular velocity of the wheel, while the other components are linked to the free-rolling motion of the rollers around their own axis. Therefore, with this type of wheel, every linear velocity of the center of the wheel is permitted. It should be underlined that, even if every linear velocity of the center of the wheel is permitted, only the components along the driving direction can be controlled. Thus, a non-singular set of unconventional wheels needs to be used to achieve omnidirectional mobility.

On the contrary, for conventional wheels, no free-sliding motion is permitted. Therefore, a generic linear velocity $\dot{\mathbf{p}}_i$ of the center of the $i$-th wheel is possible through a rotation around a vertical axis (steering motion) that aligns the driving direction with the desired linear velocity.

In the following sections, the kinematic model of the most commonly adopted omni wheels and mecanum wheels based robots is derived. Later, a particular pseudo-omnidirectional locomotion system, based on conventional wheels, is presented and analyzed.

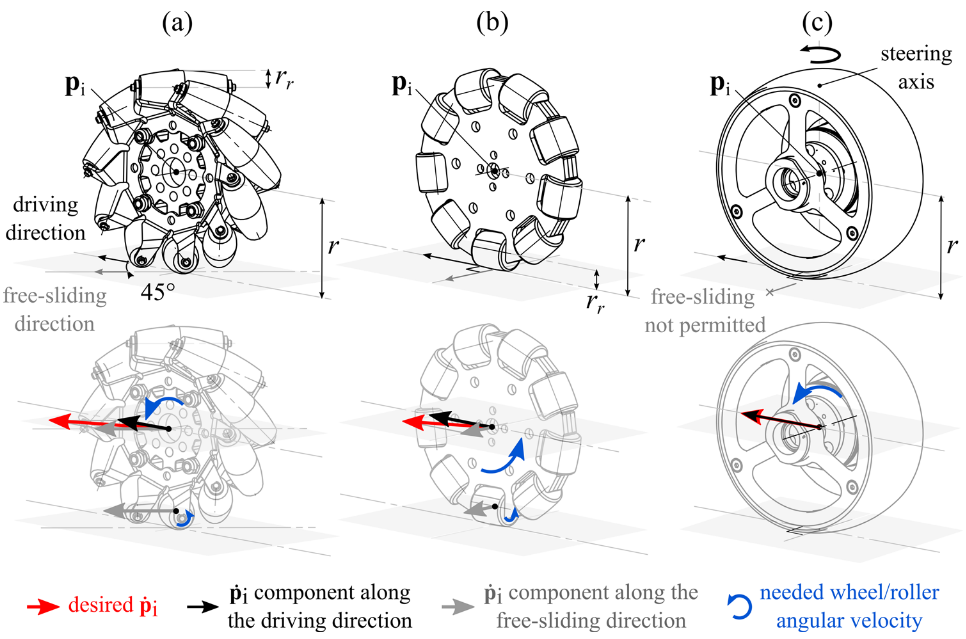

**Fig. 1** Schematic representation of the velocity analysis, under pure rolling assumption, on three types of wheels: mecanum wheel (**a**), omni wheel (**b**) and a conventional wheel (**c**)

## 2.1 Omnidirectional Wheeled Mobile Robots Modelling

To derive the kinematic model of omnidirectional wheeled mobile robots, it is essential to understand the kinematics of the non-conventional wheels adopted for these platforms. A comprehensive model for both mecanum and omni wheels has been deeply investigated in the book [91]. For a better understanding of the following sections, it is useful to recall such a kinematic model. Named $\{b\}$ the r.f. fixed to the chassis, the center of the wheel $i$ is located at ${}^b\mathbf{p}_i = [x_i, y_i]^T$ and the forward driving direction, the direction where the wheel $i$ rolls without slipping, is tilted at an angle $\phi_i$ relative to the $x$-axis of r.f. $\{b\}$. The rollers around the wheel rim allow free-sliding in a direction at an angle $\mu_i$ relative to the direction perpendicular to the driving direction, as shown in Fig. 2. For an omni wheel $\mu_i = 0^\circ$, while for a mecanum wheel $\mu_i = \pm 45^\circ$.

The linear velocity of the point ${}^b\mathbf{p}_i$ can be expressed as the sum of the driving velocity and the free sliding velocity and it can be evaluated from the body twist in the $\{b\}$ frame as:

$$ {}^b\dot{\mathbf{p}}_i = \begin{bmatrix} -y_i & 1 & 0 \\ x_i & 0 & 1 \end{bmatrix} \mathbf{V}_b \tag{1} $$

The component of ${}^b\dot{\mathbf{p}}_i$ along the driving direction, called ${}^{d_i}\dot{\mathbf{p}}_i$, can be expressed as:

$$ {}^{d_i}\dot{\mathbf{p}}_i = \begin{bmatrix} 1 & t_{\mu_i} \end{bmatrix} \begin{bmatrix} c_{\phi_i} & s_{\phi_i} \\ -s_{\phi_i} & c_{\phi_i} \end{bmatrix} \begin{bmatrix} -y_i & 1 & 0 \\ x_i & 0 & 1 \end{bmatrix} \mathbf{V}_b \tag{2} $$

Thus, the wheel driving speed $\omega_i$ can be evaluated as:

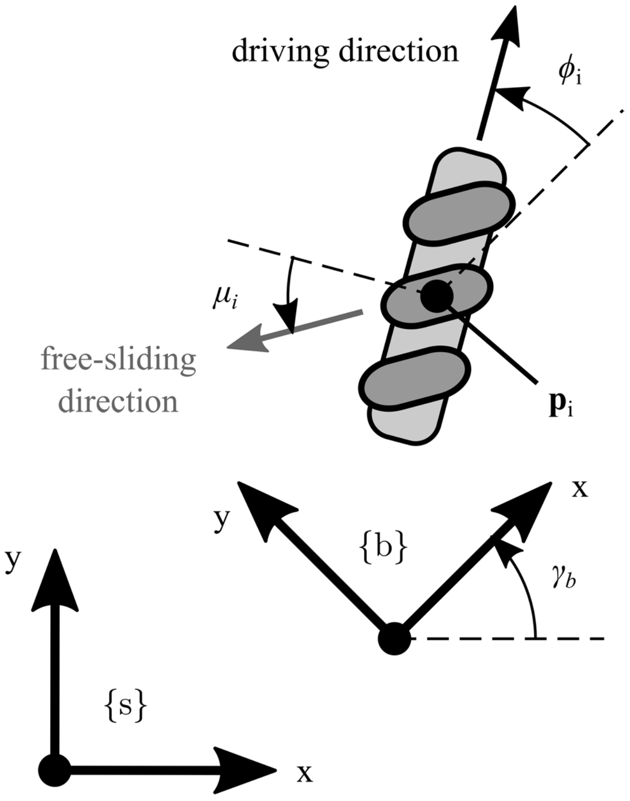

**Fig. 2** Kinematic model of a non-conventional wheel

$$ \omega_i = \frac{1}{r_i} \begin{bmatrix} 1 & t_{\mu_i} \end{bmatrix} \begin{bmatrix} c_{\phi_i} & s_{\phi_i} \\ -s_{\phi_i} & c_{\phi_i} \end{bmatrix} \begin{bmatrix} -y_i & 1 & 0 \\ x_i & 0 & 1 \end{bmatrix} \mathbf{V}_b = h_i(0)\mathbf{V}_b \tag{3} $$

where $c_\theta = \cos(\theta)$, $s_\theta = \sin(\theta)$ and $t_\theta = \tan(\theta)$.

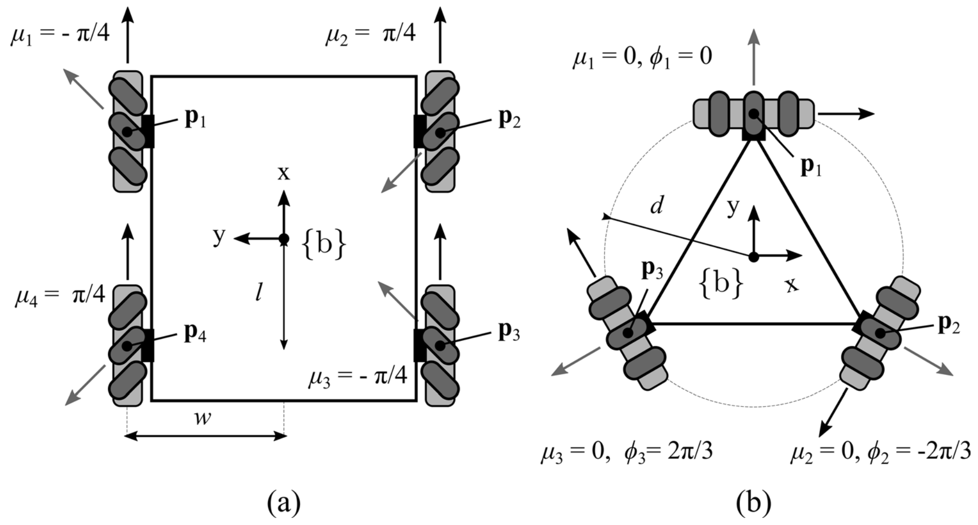

**Fig. 3** Kinematic model of a four mecanum wheel platform (a) and a three omni wheel one (b)

Given a platform with $m$ wheels, the wheel driving speed vector $\boldsymbol{\omega}$ is related to the 2D velocity twist $\mathbf{V}_b$ by the Eq. 4.

$$\boldsymbol{\omega} = \begin{bmatrix} \omega_1 \\ \vdots \\ \omega_m \end{bmatrix} = H(0)\mathbf{V}_b \qquad \text{where} \quad H(0) = \begin{bmatrix} h_1(0) \\ \vdots \\ h_m(0) \end{bmatrix} \in \mathbb{R}^{m\times 3} \tag{4}$$

The wheels' positions and headings ($\phi_i$,$x_i$,$y_i$) and their free-sliding directions $\mu_i$ must be chosen so that $H(0)$ is rank 3.

In Fig. 3, two of the most commonly adopted architectures that exploit non-conventional wheels are presented. Platform (a) is characterized by four mecanum wheels with $\phi_i = 0^\circ$, while platform (b) is composed of three omni wheels whose axes intersect at the origin of $\{b\}$ r.f.. The first architecture is used in many commercial platform like the *4WD* platform by Nexus Robot or the *youBot* by KUKA, while the latter is adopted in commercial product like the *3WD* platform by Active Robots or the one by Nexus Robot. Moreover, there are lots of application studies in the literature that make use of these types of locomotion systems [26–29].

By applying Eq. 4 to case (a) and case (b), it is possible to derive the relation between the 2D velocity twist of the platform $\mathbf{V}_b$ and the actuation vector $\boldsymbol{\omega}$. For the platforms with four mecanum wheels, this relation can be expressed in the form:

$$\boldsymbol{\omega} = \begin{bmatrix} \omega_1 \\ \omega_2 \\ \omega_3 \\ \omega_4 \end{bmatrix} = H(0)\mathbf{V}_b = \frac{1}{r}\begin{bmatrix} -l-w & 1 & -1 \\ l+w & 1 & 1 \\ l+w & 1 & -1 \\ -l-w & 1 & 1 \end{bmatrix}\begin{bmatrix} \dot{\gamma}_b \\ \dot{x}_b \\ \dot{y}_b \end{bmatrix} \tag{5}$$

while for a three omni wheels platform the relation is:

$$\boldsymbol{\omega} = \begin{bmatrix} \omega_1 \\ \omega_2 \\ \omega_3 \end{bmatrix} = H(0)\mathbf{V}_b = \frac{1}{r}\begin{bmatrix} -d & 1 & 0 \\ -d & -1/2 & -\sqrt{3}/2 \\ -d & -1/2 & \sqrt{3}/2 \end{bmatrix}\begin{bmatrix} \dot{\gamma}_b \\ \dot{x}_b \\ \dot{y}_b \end{bmatrix} \tag{6}$$

For a robot with three omni wheels, $H(0)$ matrix is square, so an arbitrary choice of wheels speeds will cause no skidding of the wheels in the driving direction. On the contrary, for a platform with four mecanum wheels the $H(0)$ matrix is not square, therefore an arbitrary choice of wheels speeds could result in skidding of the wheels in the driving direction. To avoid skidding, the driving angular speeds must be chosen on a three-dimensional surface in the four-dimensional wheel speed space.

To evaluate the inverse relation, which correlates the wheel velocities to chassis velocity twist, it is useful to adopt the pseudo-inverse $H^\dagger(0)$ of $H(0)$ matrix. For a robot with four mecanum this relation can be expressed as:

$$\begin{bmatrix} \dot{\gamma}_b \\ \dot{x}_b \\ \dot{y}_b \end{bmatrix} = H^\dagger(0)\boldsymbol{\omega} = \frac{r}{4}\begin{bmatrix} -1/(l+w) & 1/(l+w) & 1/(l+w) & -1/(l+w) \\ 1 & 1 & 1 & 1 \\ -1 & 1 & -1 & 1 \end{bmatrix}\boldsymbol{\omega} \tag{7}$$

while for a three omni wheels platform the relation is:

$$\begin{bmatrix} \dot{\gamma}_b \\ \dot{x}_b \\ \dot{y}_b \end{bmatrix} = H^{-1}(0)\boldsymbol{\omega} = \frac{r}{3}\begin{bmatrix} -1/d & -1/d & -1/d \\ 2 & -1 & -1 \\ 0 & -\sqrt{3} & \sqrt{3} \end{bmatrix}\boldsymbol{\omega} \tag{8}$$

## 2.2 Pseudo-Omnidirectional Wheeled Mobile Robots Modelling

Non-holonomic wheeled mobile robots employ conventional wheels that do not allow sideways sliding. Most of the platforms provided with conventional wheels are characterized by mobility limitations associated with the presence of fixed axis/axes of rotations. Differential drive platforms and car-like robots are widespread in the market, because they are simple to use and control. However, in some cases, their mobility limitation is not acceptable and different solutions need to be adopted. For example, it is possible to achieve pseudo-omnidirectional mobility through the adoption of two or more driving steering wheels independently actuated. The term pseudo-omnidirectional mobility is used in this paper to underline that omnidirectional mobility is achieved through an internal reconfiguration of the actuated degrees of freedom of the system. For this reason, the relation between the actuation variable and the platform velocity twist, which is presented in this section, will be configuration-dependent, unlike the one derived for the omnidirectional platform exploiting non-conventional wheels.

To achieve pseudo omnidirectional mobility in the plane of motion using conventional wheels, at least two driving steering wheels independently actuated are needed. These subsystems are also known as swerve drive systems. Some platforms adopted in robotic competitions are provided with more than two swerve drive modules to avoid dynamic problems related to castor wheels at high speed, but since two modules are enough to obtain such mobility, a two swerve drive platform is here analyzed. Moreover, the presence of more than two swerve drive modules does not change the fundamental kinematic relations, it only introduces more constraints to the model to deal with the high level of actuation redundancy.

In Fig. 4, a schematic representation of a platform with two swerve drive systems is presented. The pose of the robot in the space is defined through r.f. $\{b\}$ which lies in the middle of the segment connecting the swerve drive contact points, $\mathbf{p}_1 = [x_1, y_1]^T$ and $\mathbf{p}_2 = [x_2, y_2]^T$, with the ground. The actuation variables are two for each driving steering unit: $\delta_i$ the steering angle and $\omega_i$ the angular velocity of the wheel. The relationship between the actuation vector $\mathbf{q} = [\delta_1, \omega_1, \delta_2, \omega_2]^T$ and the platform velocity twist $\mathbf{V}_b$ can be expressed as:

$$\mathbf{V}_b = \begin{bmatrix} \dot{\gamma}_b \\ \dot{x}_b \\ \dot{y}_b \end{bmatrix} = \frac{r}{y_1 - y_2} \begin{bmatrix} -c_{\delta_1} & c_{\delta_2} \\ -y_2 c_{\delta_1} & y_1 c_{\delta_2} \\ x_1 c_{\delta_1} + (y_1 - y_2) s_{\delta_1} & -x_1 c_{\delta_2} \end{bmatrix} \begin{bmatrix} \omega_1 \\ \omega_2 \end{bmatrix} \tag{9}$$

As previously stated, the relation between the actuation variable and the velocity twist is configuration dependent: it is a function of the steering angles $\delta_1$ and $\delta_2$. Of course, an arbitrary choice of the four actuation variables could results in skidding of the wheels in the driving direction. The pure rolling condition applied to the traction wheel, which has been adopted to derive Eq. 9, leads to the following constraint:

$$\big((x_1 - x_2) c_{\delta_1} + (y_1 - y_2) s_{\delta_1}\big)\omega_1 = \big((x_1 - x_2) c_{\delta_2} + (y_1 - y_2) s_{\delta_2}\big)\omega_2 \tag{10}$$

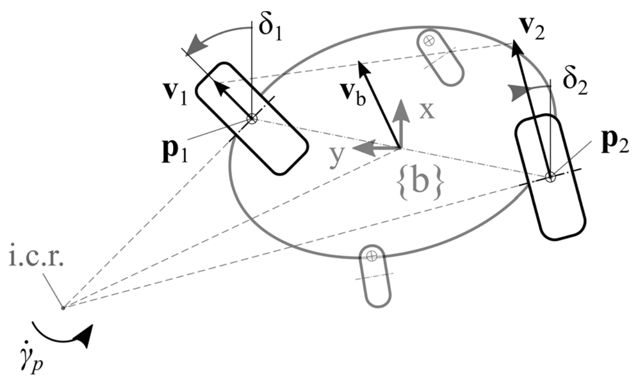

**Fig. 4** Kinematic model of a platform with two independent swerve drive systems

The Eq. 10 can be also derived from the rigid body assumption applied to the platform, because, de facto, it imposes equal velocities at the contact points $\mathbf{p}_1$ and $\mathbf{p}_2$ along the direction which connects the contact points themselves.

In the last few years, the researchers at Politecnico di Torino proposed a wheeled mobile robot, called Paquitop (which stands for *Personal Assistant Qu Italy TOrino Politecnico*) specifically conceived for home and hospital assistance of fragile subjects. The platform has been designed to obtain pseudo-omnidirectional mobility. To such an aim, the platform is suspended on four wheels: two standard off-centered passive castor wheels and two driven wheels which are also provided with a steering degree of freedom. The kinematics of the platform has been deeply studied in a previous work [92], but its kinematic architecture could be also seen as a particular case of general two swerve-drive platform scheme previously presented. The Paquitop platform is characterized by a non-symmetric footprint so that the robot is able to offer, when needed, the reduced size to pass through confined spaces. Referring to the nomenclature adopted in this work, the wheels are mounted along the longer axis of the elliptic footprint, at $\mathbf{p}_1 = [0, a]^T$ and $\mathbf{p}_2 = [0, -a]^T$. In this specific case, the Eq. 9 and the kinematic constraint Eq. 10 can be expressed as:

$$\mathbf{V}_b = \begin{bmatrix} \dot{\gamma}_b \\ \dot{x}_b \\ \dot{y}_b \end{bmatrix} = \frac{r}{2a} \begin{bmatrix} -c_{\delta_1} & c_{\delta_2} \\ a c_{\delta_1} & a c_{\delta_2} \\ a s_{\delta_1} & a s_{\delta_2} \end{bmatrix} \begin{bmatrix} \omega_1 \\ \omega_2 \end{bmatrix} \qquad \text{with } s_{\delta_1}\omega_1 = s_{\delta_2}\omega_2 \tag{11}$$

After few manipulation, it is possible to derive the velocity inverse kinematics, i.e. the relation which links the twist velocity $\mathbf{V}_b$ to the actuation vector $\mathbf{q} = [\delta_1, \omega_1, \delta_2, \omega_2]^T$.

$$\begin{bmatrix} \omega_1 \\ \omega_2 \end{bmatrix} = \frac{1}{r} \begin{bmatrix} \pm\sqrt{\left(\dot{x}_b + a\dot{\gamma}_b\right)^2 + \left(\dot{y}_b\right)^2} \\ \pm\sqrt{\left(\dot{x}_b - a\dot{\gamma}_b\right)^2 + \left(\dot{y}_b\right)^2} \end{bmatrix} \qquad \begin{bmatrix} \delta_1 \\ \delta_2 \end{bmatrix} = \begin{bmatrix} \operatorname{atan2}\left(\frac{\dot{y}_b}{r\omega_1}, \frac{\dot{x}_b + a\dot{\gamma}_b}{r\omega_1}\right) \\ \operatorname{atan2}\left(\frac{\dot{y}_b}{r\omega_2}, \frac{\dot{x}_b - a\dot{\gamma}_b}{r\omega_2}\right) \end{bmatrix} \tag{12}$$

This relation, presented in Eq. 12, has four independent solutions. It should be noticed that no solutions can be computed for the steering angles for $\omega_1 = 0$ or $\omega_2 = 0$, but if null velocity is required from one or both wheels there is no point in evaluating the corresponding steering angle.

## 3 Velocity Space Under Wheel Speed Limitation

In this section, the three platforms previously presented are compared to investigate how the traction wheels angular velocity limitation affects the achievable body twist of the platform. To effectively compare the three locomotion architectures, it is useful to adopt a dimensionless approach. Thus, the dimensionless linear velocities can be defined as $\dot{X}_b = \dot{x}_b/(r\omega_{max})$ and $\dot{Y}_b = \dot{y}_b/(r\omega_{max})$, while the yaw angular rate becomes $\dot{\Gamma}_b = (\lambda\dot{\gamma})/(r\omega_{max})$, where $\lambda$ is the geometric parameter characteristic of the locomotion architecture: for a 4WD platform $\lambda = (l + w)$, for a 3WD platform $\lambda = d$, while for a 2SWD robot, like Paquitop, $\lambda = a$. To complete this dimensionless analysis, a dimensionless wheel angular velocity is defined as $\Omega_i = \omega_i/\omega_{max}$. Using these dimensionless variables the inverse kinematics relations Eq. 5, Eq. 6 and Eq. 12 can be re-written as:

$$\begin{aligned} \text{4WD: } \rightarrow \begin{bmatrix} \Omega_1 \\ \Omega_2 \\ \Omega_3 \\ \Omega_4 \end{bmatrix} &= \begin{bmatrix} -1 & 1 & -1 \\ 1 & 1 & 1 \\ 1 & 1 & -1 \\ -1 & 1 & 1 \end{bmatrix} \begin{bmatrix} \dot{\Gamma}_b \\ \dot{X}_b \\ \dot{Y}_b \end{bmatrix} \\ \text{3WD: } \rightarrow \begin{bmatrix} \Omega_1 \\ \Omega_2 \\ \Omega_3 \end{bmatrix} &= \begin{bmatrix} -1 & 1 & 0 \\ -1 & -1/2 & -\sqrt{3}/2 \\ -1 & -1/2 & \sqrt{3}/2 \end{bmatrix} \begin{bmatrix} \dot{\Gamma}_b \\ \dot{X}_b \\ \dot{Y}_b \end{bmatrix} \end{aligned} \quad (13)$$

$$\text{2SWD: } \rightarrow \begin{bmatrix} \Omega_1 \\ \Omega_2 \end{bmatrix} = \begin{bmatrix} \pm\sqrt{(\dot{X}_b + \dot{\Gamma}_b)^2 + (\dot{Y}_b)^2} \\ \pm\sqrt{(\dot{X}_b - \dot{\Gamma}_b)^2 + (\dot{Y}_b)^2} \end{bmatrix}; \begin{bmatrix} \delta_1 \\ \delta_2 \end{bmatrix} = \begin{bmatrix} \text{atan2}\left(\frac{\dot{Y}_b}{\Omega_1}, \frac{\dot{X}_b + \dot{\Gamma}_b}{\Omega_1}\right) \\ \text{atan2}\left(\frac{\dot{Y}_b}{\Omega_2}, \frac{\dot{X}_b - \dot{\Gamma}_b}{\Omega_2}\right) \end{bmatrix}$$

In all real platforms, the driving angular velocity of the traction wheel $i$ is subjected to the bound $\|\omega_i\| \leq \omega_{max} \Leftrightarrow \|\Omega_i\| \leq 1$, i.e. $-1 \leq \Omega_i \leq 1$. For an omnidirectional robot where the relation between the actuation variables and the velocity twist is not configuration dependent, the velocity constraint $\|\omega_i\| \leq \omega_{max}$ generates two parallel constraint planes in the three-dimensional space of body-twist. The combination of the constraints applied to the $m$ wheels of the platform results in a convex three-dimensional polyhedron with $2m$ faces in which all the possible body twists $\mathbf{V}_b$ must collected. The achievable body twist space becomes more complicated for a platform with a configuration-dependent relation between the actuation variables and the velocity twist. In Figs. 5, 6 and 7, the allowed dimensionless body twist spaces for the three locomotion architectures are presented. All the set of dimensionless body twist $[\dot{\Gamma}_b, \dot{X}_b, \dot{Y}_b]^T$ must lie inside these portions of the body twist space.

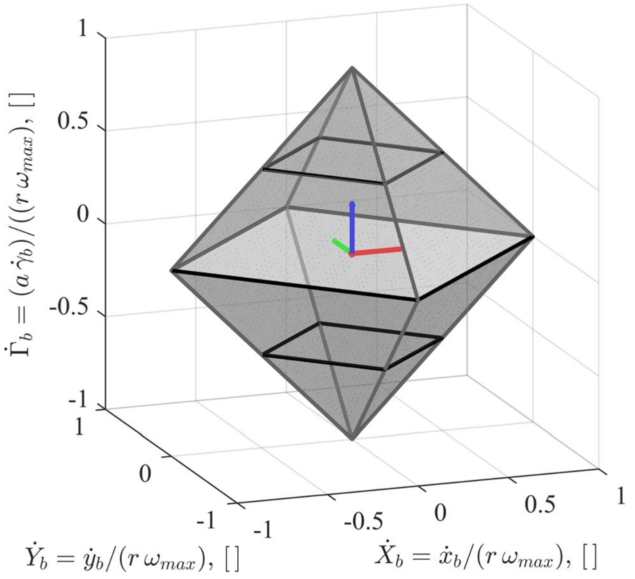

**Fig. 5** Convex three-dimensional polyhedron of allowed body twists for a 4WD omnidirectional robot

For a 4WD platform, the convex three-dimensional polyhedron is a square based octahedron. For null angular velocity $\dot{\gamma}_b$ of the platform, the linear velocity must lie inside a square with diagonals oriented along the $\dot{X}_b$ and $\dot{Y}_b$ axes and vertex at [1,0], [0,1], [−1,0] and [0,−1]. As the angular velocity absolute value $\|\dot{\Gamma}_b\|$ increases, the square is scaled down by a factor of $(1 - \dot{\Gamma}_b)$, until it collapses into a point for $\|\dot{\Gamma}_b\| = 1$.

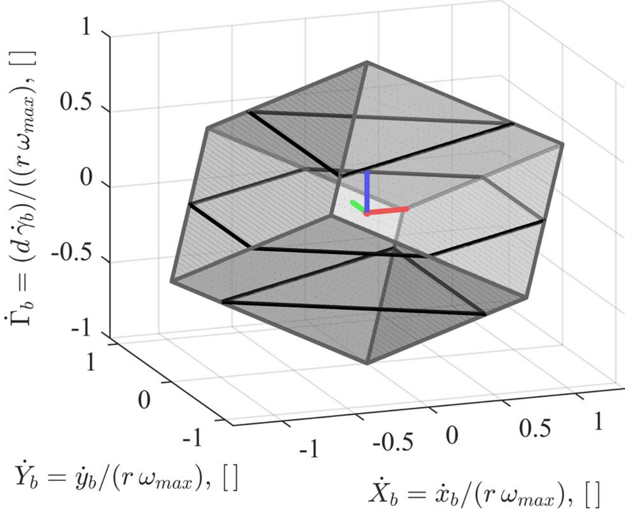

**Fig. 6** Convex three-dimensional polyhedron of allowed body twists for a 3WD omnidirectional robot

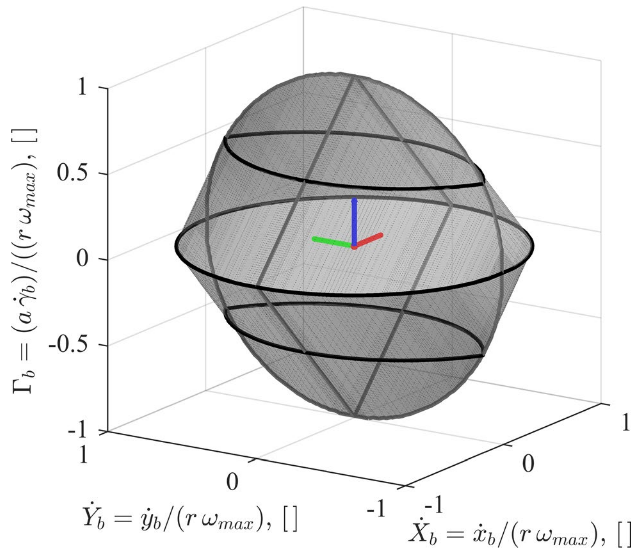

**Fig. 7** Convex three-dimensional polyhedron of allowed body twists for a 2SWD pseudo-omnidirectional robot

For a 3WD platform, the convex three-dimensional polyhedron is a parallelepiped with diagonals crossing at the origin. For null dimensionless angular velocity $\dot{\Gamma}_b$ of the platform, the linear velocity must lie inside an hexagon with vertex at $[1, \frac{\sqrt{3}}{3}]$, $[0, \frac{2\sqrt{3}}{3}]$, $[-1, \frac{\sqrt{3}}{3}]$, $[-1, -\frac{\sqrt{3}}{3}]$, $[0, -\frac{2\sqrt{3}}{3}]$ and $[1, -\frac{\sqrt{3}}{3}]$.

For a 2SWD platform, like Paquitop, the convex three-dimensional space of the allowed body twist is a particular solid with unit circular cross-sections on the $\dot{X}_b - \dot{Y}_b$ and $\dot{Y}_b - \dot{\Gamma}_b$ planes and a unit square cross-section on the $\dot{X}_b - \dot{\Gamma}_b$ plane. This velocity limitation associated with the longitudinal speed $\dot{X}_b$ is related to the fact that while the platform is moving along the longitudinal direction a yaw angular rate can be achieved only with differential drive strategy. Therefore, the outer wheel reaches the maximum speed for a value of $\dot{X}_b$ and $\dot{\Gamma}_b$ lower than 1. On the contrary, when the platform is moving along the transverse direction a yaw angular rate is achieved exploiting the steering degrees of actuation, thus no limitation associated with differential drive steering occurs.

With this dimensionless analysis, it is also possible to underline the importance of the geometric parameter of the platform, in particular the wheel radius $r$ and the geometric parameter $\lambda$. The wheel's radius $r$ acts as a scaling factor for the convex velocity constraints previously presented, while the geometric parameter $\lambda$ stretches the allowed velocity space along the $\dot{\Gamma}_b$ axis. An increment of the wheel radius results in an increment of the maximum velocity allowed in every direction, while an increment in the geometric parameter $\lambda$ results in a contraction of the velocity space along the $\dot{\Gamma}_b$ axis.

A comparison among the allowed body twists resulting from the three locomotion systems is presented in Fig. 8. In particular, Fig. 8 (a) shows the velocity constraints when the platforms' motion is pure translational. In this condition, the linear velocity of a 4WD platform must lie inside a unit square with diagonals oriented along the $\dot{X}_b$ and $\dot{Y}_b$ axes, for a 3WD platform the velocity constraint is an hexagon of vertex $[1, \frac{\sqrt{3}}{3}]$, $[0, \frac{2\sqrt{3}}{3}]$, $[-1, \frac{\sqrt{3}}{3}]$, $[-1, -\frac{\sqrt{3}}{3}]$, $[0, -\frac{2\sqrt{3}}{3}]$ and $[1, -\frac{\sqrt{3}}{3}]$, while for a 2SWD robot like Paquitop the pure

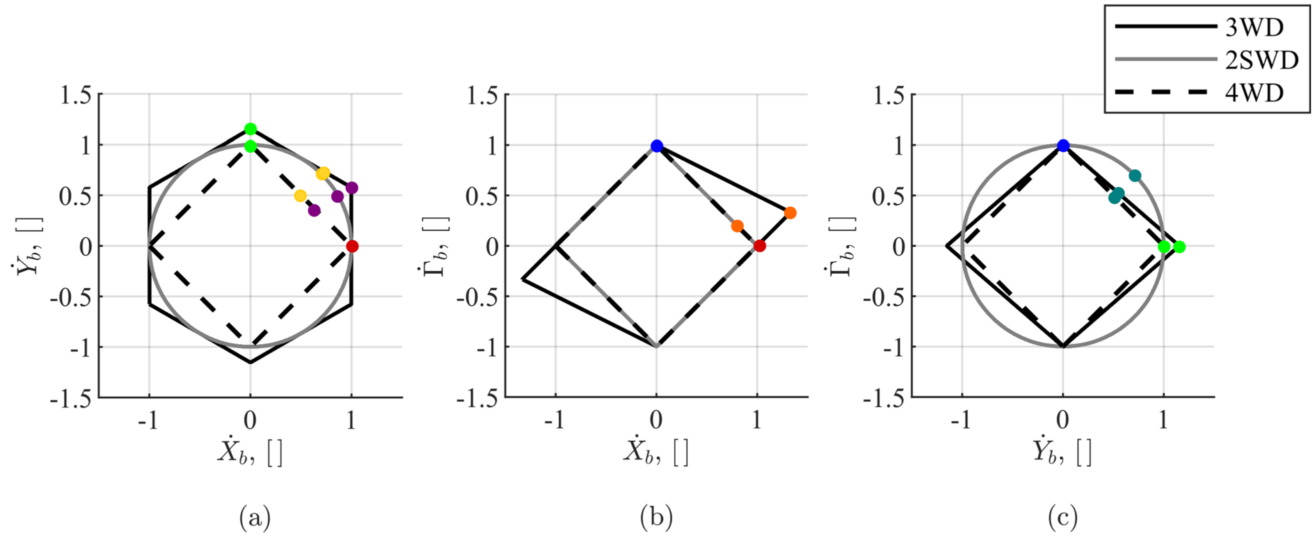

**Fig. 8** Comparison among the velocity constraints of a 4WD, a 3WD and a 2SWD robot on the planes: $\dot{\Gamma}_b = 0$, (**a**), $\dot{Y}_b = 0$, (**b**), $\dot{X}_b = 0$, (**c**). The color points specify the velocity states related to the case studies presented in Table 2 and analysed in the rest of this chapter: red ($x$.1), green ($x$.2), blue ($x$.3), yellow ($x$.4), purple ($x$.5), orange ($x$.6) and dark-green ($x$.7). It should be underlined that case ($x$.8) cannot be represented in this sections becuase the velocity state does not lie in any of these planes

**Table 2** Detailed description of the analysed particular cases

| Case | $V_b = [\dot{\Gamma}_b \quad \dot{X}_b \quad \dot{Y}_b]^T$ | Motion Description | Color |
|---|---|---|---|
| (a.1) | $[0 \quad 1 \quad 0]^T$ | | |
| (b.1) | $[0 \quad 1 \quad 0]^T$ | Pure translation along $x$ axis | Red |
| (c.1) | $[0 \quad 1 \quad 0]^T$ | | |
| (a.2) | $[0 \quad 0 \quad 1]^T$ | | |
| (b.2) | $[0 \quad 0 \quad \frac{2\sqrt{3}}{3}]^T$ | Pure translation along $y$ axis | Green |
| (c.2) | $[0 \quad 0 \quad 1]^T$ | | |
| (a.3) | $[1 \quad 0 \quad 0]^T$ | | |
| (b.3) | $[1 \quad 0 \quad 0]^T$ | Pure rotation around $z$ axis | Blue |
| (c.3) | $[1 \quad 0 \quad 0]^T$ | | |
| (a.4) | $[0 \quad 1/2 \quad 1/2]^T$ | | |
| (b.4) | $[0 \quad \sqrt{3}-1 \quad \sqrt{3}-1]^T$ | Pure translation at 45° wrt $x$ axis | Yellow |
| (c.4) | $[0 \quad \frac{\sqrt{2}}{2} \quad \frac{\sqrt{2}}{2}]^T$ | | |
| (a.5) | $[0 \quad \frac{3-\sqrt{3}}{2} \quad \frac{\sqrt{3}-1}{2}]^T$ | | |
| (b.5) | $[0 \quad 1 \quad \frac{\sqrt{3}}{3}]^T$ | Pure translation at 30° wrt $x$ axis | Purple |
| (c.5) | $[0 \quad \frac{\sqrt{3}}{2} \quad \frac{1}{2}]^T$ | | |
| (a.6) | $[1/5 \quad 4/5 \quad 0]^T$ | | |
| (b.6) | $[1/3 \quad 4/3 \quad 0]^T$ | Roto-translation $\dot{X}_b = 4\dot{\Gamma}_b,\ \dot{Y}_b = 0$ | Orange |
| (c.6) | $[1/5 \quad 4/5 \quad 0]^T$ | | |
| (a.7) | $[1/2 \quad 0 \quad 1/2]^T$ | | |
| (b.7) | $[\frac{\sqrt{3}-1}{2} \quad 0 \quad \frac{\sqrt{3}-1}{2}]^T$ | Roto-translation $\dot{Y}_b = \dot{\Gamma}_b,\ \dot{X}_b = 0$ | Dark green |
| (c.7) | $[\frac{\sqrt{2}}{2} \quad 0 \quad \frac{\sqrt{2}}{2}]^T$ | | |
| (a.8) | | | |
| (b.8) | – | Generic omnidirectional motion | Pink |
| (c.8) | | | |

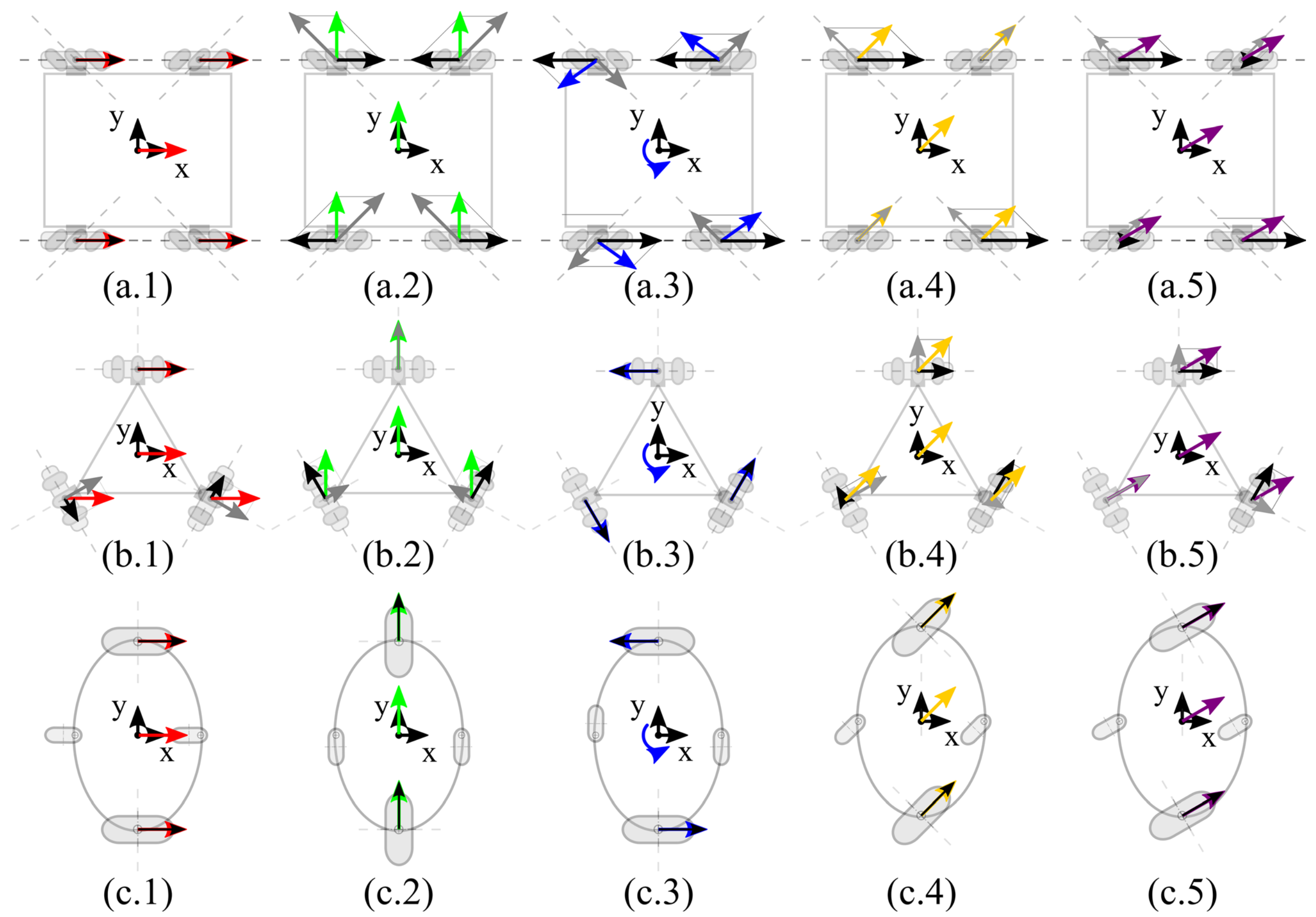

**Fig. 9** Velocity analysis of the three architectures (4WD (a.*i*), 3WD (b.*i*) and (2SWD(c.*i*)) in specific acts of motion: pure translation along the *x* axis (*x*.1), pure translation along the *y* axis (*x*.2), pure rotation around the *z* axis (*x*.3), pure translation at 45° relative to the *x* axis (*x*.4) and pure translation at 30° relative to the *x* axis (*x*.5)

translational velocity constraint is a unit circle. Therefore, the translational mobility of a 4WD architecture is more limited with respect to the other architectures. The maximum linear velocity magnitude for a 2SWD robot is equal to 1 in every direction, while for a 3WD platform the maximum dimensionless velocity is $\frac{2\sqrt{3}}{3}$ and it can be achieved along six directions equally spaced at $\pi/3$ radians. In Fig. 8 (b) and (c) the kinematic constraints resulting on the planes $\dot{Y}_b = 0$ and $\dot{X}_b = 0$ are represented. When the transverse velocity is equal to zero ($\dot{Y}_b = 0$), the 4WD and the 2SWD architectures result in the same unit square limitation which can be associated with the effects of the differential steering strategy. At the contrary the 3WD platform results in a quadrilateral shape, with vertex at $[-\frac{4}{3}, -\frac{1}{3}]$, $[0, -1]$, $[\frac{4}{3}, \frac{1}{3}]$ and $[0,1]$. When the longitudinal velocity is equal to zero ($\dot{X}_b = 0$), the 4WD and the 3WD architectures are characterized by similar constraints: for a 4WD robot the map is the previously described unit square, while for a 3WD robot the map is a rhombus like map with vertex at $[-\frac{\sqrt{3}}{3}, 0]$, $[0,1]$, $[\frac{\sqrt{3}}{3}, 0]$ and $[0, -1]$. At the contrary, the 2SWD platform velocities must lie inside a unit circle associated with the exploiting of the steering degrees of freedom.

It should be underlined that the comparison here presented depends on the reference frames' definition. Therefore, these dimensionless body twist constraint shapes can be rearranged in different manners by changing the orientation of the reference frames $\{b\}$. Nevertheless, it can be stated that a wheels' speed limitation in a 4WD architecture results in major velocity constraints that are equal on the three orthogonal planes $\dot{X}_b = 0$, $\dot{Y}_b = 0$ and $\dot{\Gamma}_b = 0$. Moreover, a 3WD platform is characterized by the highest values of the body twist achievable, but also by a highly non-symmetrical behavior. Interesting kinematic properties can be appreciated for a 2SWD architecture when the motion strategy exploits the independent steering angles, while higher velocity limitations occur when differential steering is needed.

From a practical point of view, a comparison among the three locomotion strategies can be made by evaluating the most relevant geometric parameters, $r$ and $\lambda$, needed to

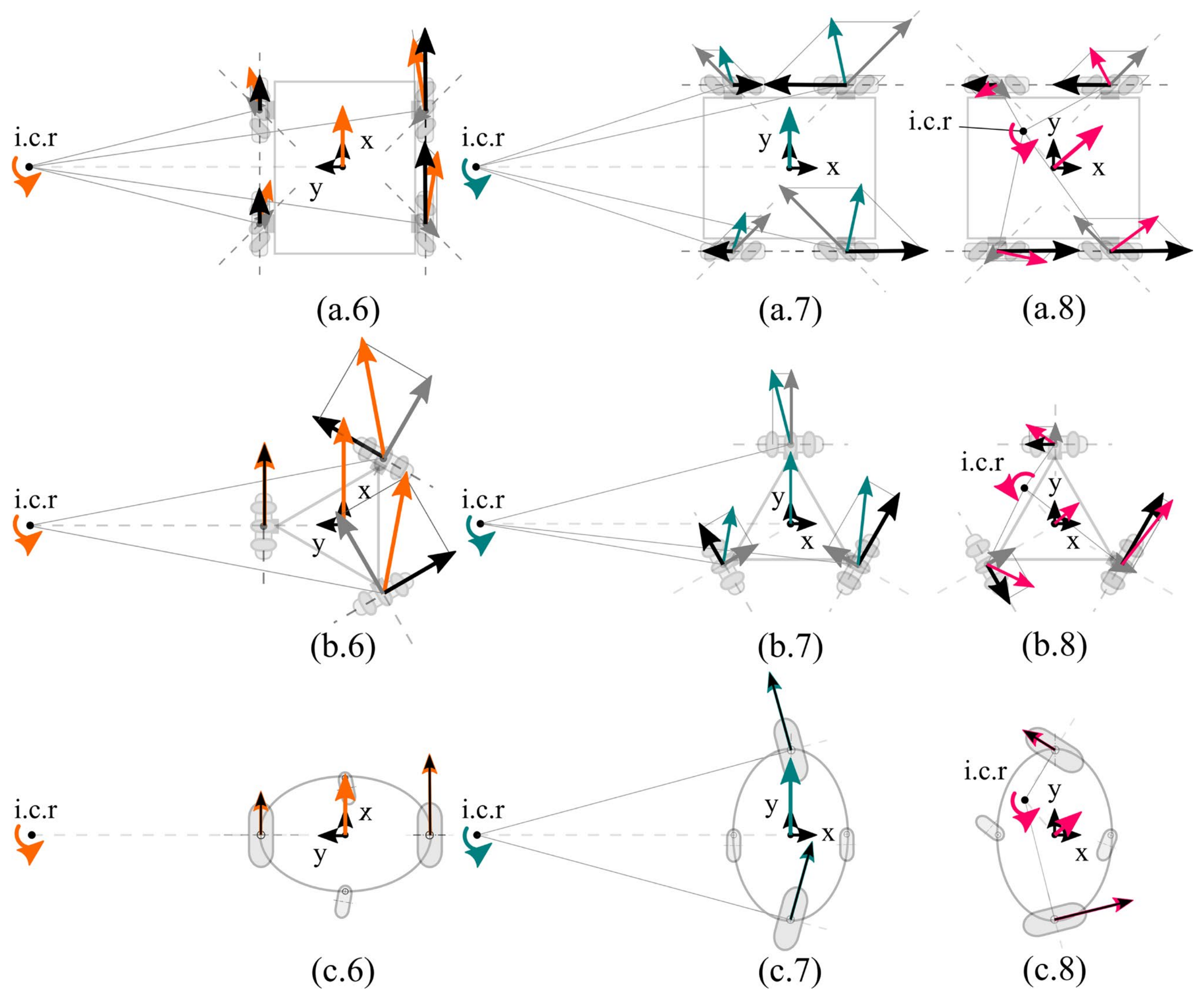

**Fig. 10** Velocity analysis of the three architectures (4WD (a.$i$), 3WD (b.$i$) and (2SWD(c.$i$)) during three acts of motion: $\dot{X}_b/\dot{\Gamma}_b = 4$ ($x$.6), $\dot{Y}_b/\dot{\Gamma}_b = 1$ ($x$.7), generic configuration ($x$.8)

address the application-related velocity requirements. Let's consider the following requirements:

1. pure translational motion with a maximum velocity absolute value $v_{max} = 5.0 km/h$ in all directions;
2. 2D roto-translational motion with a maximum linear velocity absolute value of $85\% v_{max}$ and a maximum angular velocity absolute value $\dot{\gamma}_{max} = 30 rpm$;

Let's assume that all the wheel speeds are limited by a maximum value $\omega_{max} = 120 rpm$. The first application requirement translates into a needed radius $r_{min} = v_{max}/\omega_{max} = 22.1 cm$ for the 3WD and 2SWD platform and $r_{min} = \sqrt{2} v_{max}/\omega_{max} = 31.3 cm$ for the 4WD platform. In other words, to achieve the same linear velocity in all directions during a pure translational motion, the 4WD wheels need to be 41% bigger than the other platform wheels. To evaluate the geometric parameter $\lambda$ that enables the platform to perform the 2D motion specified by the second requirement, firstly the maximum $\dot{\Gamma}_b$ allowed at a certain linear adimensional velocity is evaluated through Fig. 8 (c). Then, the $\lambda$ parameter can be evaluated through the definition of the angular adimensional velocity. It results that for a 4WD platform $\lambda = (w + l) = 54.3 cm$, for a 3WD platform $\lambda = d = 47.6 cm$, while for a 2SWD platform $\lambda = a = 17.7 cm$.

To better understand how the different locomotion systems provide such mobility, some particular cases are analyzed, a detailed desciption is presented in Table 2. In Fig. 9, a velocity analysis is performed for the three architectures under five body twist conditions. Using the same color code, these velocity states are underlined also in Fig. 8. The cases ($x$.1), ($x$.2) and ($x$.3) are characterized by a velocity vector aligned with the coordinated axes, while the cases ($x$.4) and ($x$.5) present pure translation motion. The goal of this analysis is to underline the relationship between the desired body twist of the platform and the attachment points velocity $\dot{\mathbf{p}}_i$, with particular attention to the components of $\dot{\mathbf{p}}_i$ along with the driving and the free-sliding directions, represented respectively by black and gray arrows. As previously explained at the beginning of Section 2, this velocity of the attachment point $\mathbf{p}_i$ is then related to the angular velocity of the wheel around its own axis, and the angular velocity of the roller is in contact with the ground. From this analysis, it is possible to understand which wheel(s) is(are) responsible for the velocity limitation of the platform during the execution of a specific motion. For example, in case ($x$.4) the maximum linear velocity achievable with the 4WD platform is due to the angular velocity saturation of the wheels 2 and 4, while a null velocity is required by the other two wheels. Similarly, the maximum linear velocity along the $x$ axis, case ($x$.1), achievable with a 3WD system is related to the maximum velocity of the first wheel, while a lower speed is required by the other two wheels.

Moreover, a deep analysis of the velocity conditions presented in Fig. 9 can be made to evaluate the component of the attachment points velocity $\dot{\mathbf{p}}_i$ along the free-sliding direction. This component of the velocity corresponds to the uncontrolled angular velocity of the roller in contact with the ground around its own axis. Expect in case (a.1) for a 4WD architecture and in case (b.3) for a 3WD platform, relevant velocities along the free-sliding directions are required, thus the rollers must rotate at high speed. In this sense, the construction quality of the unconventional wheels plays a key role in the performance of the platform in terms of vibrations and positioning accuracy, as presented by Adamov et Al. in 2018 [47]. Moreover, the reduced size of the roller, which is related to the relative curvature at the contact point can strongly affect the navigation in presence of small obstacles and unevenness of the ground.

To complete this velocity analysis, it is useful to study three additional cases where a roto-translation is desired, Fig. 10. In case ($x$.6), where the instantaneous center of rotation (i.c.r.) lies on the $y$ axis of $\{b\}$, the 4WD and the 2SWD system are characterized by velocity limitations related to the speed saturation on the outer wheels (in this case the ones on the right side), while the 3WD architecture is capable of higher velocity in this configuration due to its kinematic architecture. In case ($x$.7), where the instantaneous center of rotation (i.c.r.) lies on the $x$ axis of $\{b\}$, the 3WD and 4WD architecture are limited in terms of maximum velocity, while the 2SWD locomotion system properly exploits the steering degrees of actuation to achieve higher velocities. The last case, ($x$.8), represents a general act of motion and it has been included to better underline how the different locomotion architectures can achieve omnidirectional mobility. For example, the 2SWD system can exhibit a generic body twist with different values of the steering angles.

## 4 Conclusion

After a brief introduction on the social factors that have been encouraged the development of mobile robots in the last years, a detailed description of the wheeled mobile robots (WMR) is presented. The most commonly adopted solutions in terms of locomotion systems, perception sensors and approaches, control strategies, and navigation algorithms are described. Later, the paper provides kinematic models for three types of omnidirectional platforms: a four mecanum wheels based robot, a three omni wheels locomotion system and a two swerve drive platform. In the field of wheeled mobile robots, all applications are characterized by wheel speed limitations. Those limitations affect in different ways the achievable platform body twists based on the specific locomotion system adopted. To compare the three architectures, in chapter Section 3 a dimensionless approach is introduced. For the different architectures, the allowed velocity spaces are described. The different body twist constraints are compared and specific cases are presented in order to better understand the velocity state during certain acts of motion. Through this analysis, the paper intended to provide a valid tool to guide the selection of an appropriate mobile platform or the designing process of a custom solution.

Based on the results of this analysis, presented in chapter Section 3, the 3WD locomotion system mobility seems less affected by the wheel speed limitation, even if, in most of the cases, one or even two motors are not exploited, which results in a higher torque requirement for the actuation system. Moreover, it has been proved that high angular velocities of the passive rollers are needed for both omni and mecanum wheels. Therefore, these platforms will be characterized by lower efficiency and a higher level of vibration, strongly related to the construction quality of the wheels. On the contrary, the 2SWD locomotion system provides the platform with high performance, in presence of wheel speed limitation, when the two locomotion units have parallel, yet non-coincident, axes of rotations, e.g. during pure translations or roto-translation with zero longitudinal linear velocity, i.e. a 2D roto-translational motion. However, the mobility of this architecture is limited when differential drive steering is needed. In conclusion, and from a pure kinematics point of view, a 3WD platform is more suitable in applications where a generic motion is frequently required, a 4WD robot is more appropriate when there is a preferential direction of motion, while the 2SWD architecture can be better exploited when pure translations or high-speed 2D roto-translational motions are required. Finally, it should be underlined that, because of the presence of small rollers in contact with the ground, the adoption of 4WD and 3WD robots is more appropriate for indoor applications with a low level of ground unevenness.

**Author Contributions** All authors contributed to the study conception and design. Material preparation, data collection and analysis were performed by Luigi Tagliavini and Giuseppe Quaglia. The first draft of the manuscript was written by Luigi Tagliavini and all authors commented on previous versions of the manuscript. All authors read and approved the final manuscript.

**Funding** Open access funding provided by Politecnico di Torino within the CRUI-CARE Agreement. The authors declare that no funds, grants, or other support were received during the preparation of this manuscript.

## Declarations

**Ethics Approval** The research does not involve human participants, their data or biological material and it does not involve animals.

**Conflict of Interests** The authors have no relevant financial or non-financial interests to disclose.

**Open Access** This article is licensed under a Creative Commons Attribution 4.0 International License, which permits use, sharing, adaptation, distribution and reproduction in any medium or format, as long as you give appropriate credit to the original author(s) and the source, provide a link to the Creative Commons licence, and indicate if changes were made. The images or other third party material in this article are included in the article's Creative Commons licence, unless indicated otherwise in a credit line to the material. If material is not included in the article's Creative Commons licence and your intended use is not permitted by statutory regulation or exceeds the permitted use, you will need to obtain permission directly from the copyright holder. To view a copy of this licence, visit http://creativecommons.org/licenses/by/4.0/.

## Data Availability

Data sharing not applicable to this article as no datasets were generated or analysed during the current study
